# Effect of fluoride varnish with added casein phosphopeptide-amorphous calcium phosphate on the acid resistance of the primary enamel

**DOI:** 10.1186/s12903-016-0299-4

**Published:** 2016-09-26

**Authors:** Nuray Tuloglu, Sule Bayrak, Emine Sen Tunc, Fusun Ozer

**Affiliations:** 1 Department of Pediatric Dentistry, Faculty of Dentistry, Eskisehir Osmangazi University, 26480 Eskisehir, Turkey; 2 Department of Pediatric Dentistry, Faculty of Dentistry, Ondokuz Mayıs University, Samsun, Turkey; 3 Department of Preventive and Restorative Sciences, School of Dental Medicine, University of Pennsylvania, Philadelphia, USA

**Keywords:** Acid resistance, CPP-ACP, Enamel, Fluoride, Varnish

## Abstract

**Background:**

This study aimed to investigate the effects of a fluoride varnish with added Casein Phosphopeptide-Amorphous Calcium Phosphate (CPP-ACP) treatments on acid resistance of primary teeth enamel.

**Methods:**

Enamel specimens obtained from 40 primary incisors (for surface microhardness testing) and 40 primary molars (for demineralization depth measurement) were randomly divided into four groups (*n* = 10 incisors and 10 molars) each according to surface treatment: no treatment (control), MI varnish (1–8 % sodium fluoride and 1–5 % CPP-ACP), Clinpro White (1–5 % sodium fluoride and <5 % modified tricalcium phosphate), Duraphat (<5 % sodium fluoride). Specimens were stored for 24 h in a moist environment. After varnish residues were removed, specimens were subjected to pH cycling. The effects of fluoride varnishes were evaluated according to surface microhardness, lesion depth and structural changes. Results were analyzed by ANOVA and Tukey’s tests.

**Results:**

The lowest changes in surface microhardness and lesion depth occurred in MI varnish group, followed by the Clinpro White, Duraphat and no treatment (control) group (for percentage of loss surface microhardness −20.80, −34.60, −57.80 and −73.40; for lesion depth values 23.60 μm ± 3.36, 29.85 μm ± 3.27, 40.37 μm ± 3.41 and 54.56 μm ± 4.16, respectively). Statistically significant differences in both surface microhardness and lesion depth were observed among all groups (*P* < 0.05).

**Conclusions:**

Within the limitations of this in vitro study, fluoride varnish containing CPP-ACP was more effective in increasing the acid resistance of primary enamel than other fluoride varnishes. However, further clinical research is needed to confirm these in vitro results.

## Background

Dental caries is a chronic, multifactorial, transmissible infectious disease caused by acids from bacterial metabolism diffusing into enamel and dentine. While caries is a highly preventable disease that has witnessed a decline in most developed countries in recent years, it continues to remain a major public health problem, especially among young children [1].

In early stages of caries process, the fermentation of carbohydrates by bacteria decreases local pH levels below the critical value (<5.5) and results in subsurface dissolution of the mineral of the enamel [1, 2]. Therefore, one of the key factors in preventing dental caries has been recognized to be increasing the resistance of teeth to acid encouraging the development of remineralization mechanisms on the enamel surface [2]. Although many strategies have been developed for maintenance of the integrity of the enamel surface, the application of fluoride agents in various forms is a proven approach that strengthens the enamel structure, making it less susceptible to demineralization by the formation of stronger fluorapatite crystals and enhancing enamel remineralization [3–5].

Fluoride varnishes are believed to promote the formation of intraoral fluoride reservoirs through the formation of calcium fluoride [6]. Since the formation of these reservoirs is limited by the availability of calcium and fluoride ions [6], calcium ions have been added to fluoride varnishes in an attempt to increase the retention of both fluoride and calcium ions in the oral environment. Moreover, given that the remineralization potential of saliva is also limited by the amount of available calcium [7], the addition of calcium and phosphate ions from sources such as varnishes can enhance the remineralization of early lesions. Hence, many manufacturers have attempted to further improve the efficacy of fluoride varnishes through the addition of calcium and inorganic phosphate ions. Recently, more advanced fluoride varnishes with added casein phosphopeptide-amorphous calcium phosphate (CPP-ACP) have been developed [8]. Topically administered CPP-ACP buffers free calcium and phosphate ion activity, maintaining a state of supersaturation with respect to tooth enamel that helps prevent demineralization and facilitate remineralization [7–10].

Numerous reports have been published on the anticariogenic activity of CPP-ACP paste/solution [9–13] and the synergistic effect of CPP-ACP solution and fluoride [9, 14]. However, to date, not a single study has been published evaluating the effects of fluoride varnish with added CPP-ACP on the acid resistance of primary-tooth enamel. Therefore, the present study investigated the effects of fluoride varnish with added CPP-ACP treatments on acid resistance of primary teeth enamel and compared the results with those of other fluoride varnishes.

## Methods

### Preparation of enamel specimens

This study was approved by The Clinical Research Ethics Council of the Ondokuz Mayıs University (approval number: 2015/488), and all procedures were conducted in accordance with the 1975 Helsinki Declaration (as revised in 2008). A total of 80 sound extracted for orthodontic reasons or naturally exfoliated human primary incisor and molar teeth were obtained from the 6–10 year-old children. Before the extraction, the patients/parents were informed about the use of their teeth for research purposes and consent was obtained. Teeth were polished with pumice to remove any surface debris or contaminants and were stored in 0.1 % thymol solution at room temperature prior to the experiment. Teeth were examined under a stereomicroscope at ×40 magnification, and those with cracks, stains or white spot lesion were excluded. Then, the teeth roots were removed.

Surface microhardness testing was performed using the 40 prepared incisor teeth. An area of enamel approximately 2 mm × 2 mm was cut from the buccal surface of each incisor and embedded in an acrylic resin cylinder with the enamel surface exposed. Specimens were ground under running water using a polishing machine with 600- and 1200-grit silicon-carbide papers to create standardized flat surfaces [15].

Demineralization depth testing was performed using the 40 prepared molar teeth [16–18]. After sealing the apices with wax, specimens were covered with a thin coat of acid-resistant nail varnish, leaving an exposed window of enamel approximately 2 mm × 2 mm in the center of each buccal surface.

### Treatment protocol and pH cycling

Three different fluoride varnishes were evaluated: MI Varnish (GC, Tokyo, Japan), Clinpro White (3M Espe, MN, USA) and Duraphat (Colgate-Palmolive, NSW, Australia). Details of the varnishes are represented in Table 1.

**Table 1:** Varnishes used in this study

Varnish	Content	Manufacturer	Lot Number	Source
MI	30–50 % polyvinyl acetate, 10–30 % hydrogenated rosin, 20–30 % ethanol, 1–8 % sodium fluoride, 1–5 % CPP-ACP, 1–5 % silicon dioxide	GC, Tokyo, Japan	141009A	MSDS
Clinpro White	30–75 % pentaerythritol glycerol ester of colophony resin, 10–15 % n-hexane, 1–15 % ethyl alcohol, 1–5 % sodium fluoride, 1–5 % flavour enhancer,1–5 % thickener, 1–5 % food grade flavour, <5 % modified tricalcium phosphate	3 M Espe, MN, USA	N545905	MSDS
Duraphat	10- < 40 % colophonium, 10- < 30 % ethanol, <5 % sodium fluoride, <1 % saccharin, <1 % isoamyl acetate, other ingredients	Colgate-Palmolive, NSW, Australia	BB2LX	MSDS

Both 40 incisor and 40 molar specimens were randomly assigned to 1 of 4 surface-treatment groups:

- Group 1 (*n* = 10 incisors and 10 molars): No treatment (control group),
- Group 2 (*n* = 10 incisors and 10 molars): MI Varnish,
- Group 3 (*n* = 10 incisors and 10 molars): Clinpro White Varnish,
- Group 4 (*n* = 10 incisors and 10 molars): Duraphat Varnish.

The fluoride varnishes were applied according to the manufacturers’ instructions and the specimens were stored for 24 h in a moist environment [4, 19]. After varnish residues were carefully removed by surgical blade and cotton swabs soaked in acetone and washed with deionized water for 1 min [19, 20], specimens were subjected to pH cycling.

This study performed pH cycling model according to ten Cate and Duijsters [21] in order to simulate the caries process. Enamel specimens were individually immersed in a demineralizing solution (2.20 mmol/L calcium chloride, 2.20 mmol/L monosodium phosphate, 1 mol/L potassium hydroxide and 0.05 mol/L acetic acid; pH 4.4) for 6 h, rinsed with distilled water for 10 s, and gently dried with absorbent paper. Next, specimens were individually immersed in a remineralizing solution (1.5 mmol/L calcium chloride, 0.9 mmol/L monosodium phosphate, 150 mmol/L potassium chloride; pH 7.0) for 18 h. All procedures were carried out at 37 °C. Demineralizing and remineralizing solutions were renewed daily, and cycling was repeated for 7 days [22]. At the end of pH cycling, all specimens were ultrasonically cleaned for 15 min.

### Surface microhardness measurements

Enamel surface microhardness was measured before treatment (baseline) and after pH cycling using a Vickers microhardness tester (HM112, Mitutoyo Corporation, Tokyo, Japan) with a load of 100 g. Three indentations at distances of 100 μm from each other, were performed in the center of enamel specimens, the average was calculated and recorded as the Vickers microhardness number (VHN) for each specimen. Baseline (VHN1) and post-pH cycling (VHN2) Vickers microhardness numbers were used to calculate the percentage of loss surface microhardness (%VHN) following acid exposure using the formula: 100× (VHN2-VHN1) ⁄ VHN1 [20].

### Lesion depth measurements

Specimens were sectioned through the center of the lesions using a diamond saw with water cooling and polished with 600- and 1200-grit abrasive paper under water cooling to obtain sections of 100 ± 20 μm thicknesses. The sections were examined under a polarized light microscope (PLM) (DM LM, Leica Microsystems, Wetzlar, Germany) using a digital camera to obtain images at ×20 magnification. Measurements of demineralized zones were performed using image analysis software (Image Pro-Plus 6.0; Media Cybernetics, Rockville, MD, USA). For each section, the demineralized area was measured (μm) 3 times, and the average was calculated and recorded as the lesion depth for that specimen.

### Evaluation of structural changes

An enamel specimen from each group was randomly selected for evaluation of structural changes using Scanning Electron Microscopy (SEM) (Jeol JSM-5600LV, Japan). Enamel specimens were coated with a thin layer of gold. The specimens were then microscopically evaluated under ×2000 magnification at 20 kV.

### Sample size calculation and statistical analysis

The final sample size was decided after preliminary testing of 5 samples from each group. A power analysis applied to the preliminary data showed that a sample size of 10 would appropriately discriminate between the groups at a significance level of *p* < 0.05 and a power of 0.8.

One way ANOVA was used to identify significant differences (*P* < 0.05) in %VHN and demineralization depth among the groups. Post-hoc comparisons were made using the Tukey’s test. Differences between baseline and post-pH cycling mean enamel surface microhardness values were analyzed using paired t-tests. The level of significance was set at *P* < 0.05. All statistical analysis was performed using SPSS for Windows, Version 12.0.1 (SPSS Inc, Chicago, IL, USA).

## Results

Table 2 shows the baseline and post-pH cycling VHN of the groups. Baseline surface microhardness measurements ranged from 350.00 VHN to 387.20 VHN, with no statistically significant differences among the groups (*P* > 0.05). Post-pH cycling surface microhardness measurements ranged from 92.00 VHN to 279.01 VHN. The lowest changes in surface microhardness occurred in MI varnish group, followed by the Clinpro White, Duraphat and no treatment (control) group. Statistically significant differences were observed among all groups (*P* < 0.05) (Table 2).

**Table 2:** Mean values and standard deviation of the baseline (VHN1) and post-pH cycling (VHN2) surface microhardness, and mean values for percentage of surface microhardness loss (%VHN) after treatment and pH cycling

Group	VHN1	VHN2	%VHN
No treatment (control)	350.00 ± 38.64^A^	92.00 ± 24.29^B^	−73.40^d^
MI Varnish	353.30 ± 30.25^A^	279.01 ± 27.13^B^	−20.80^a^
Clinpro White	376.40 ± 36.74^A^	245.20 ± 28.91^B^	−34.60^b^
Duraphat	387.20 ± 31.12^A^	163.00 ± 27.92^B^	−57.80^c^

Different uppercase letters indicate significant difference between baseline and post- pH cycling surface microhardness values of each varnish (paired-*T* test, *P* < 0.05, *n* = 10 incisors in each group). Different lowercase letters indicate significant differences among in the %VHN between groups (one-way ANOVA and Tukey’s test, *P* < 0.05, *n* = 10 incisors in each group)

**Fig. 1:**
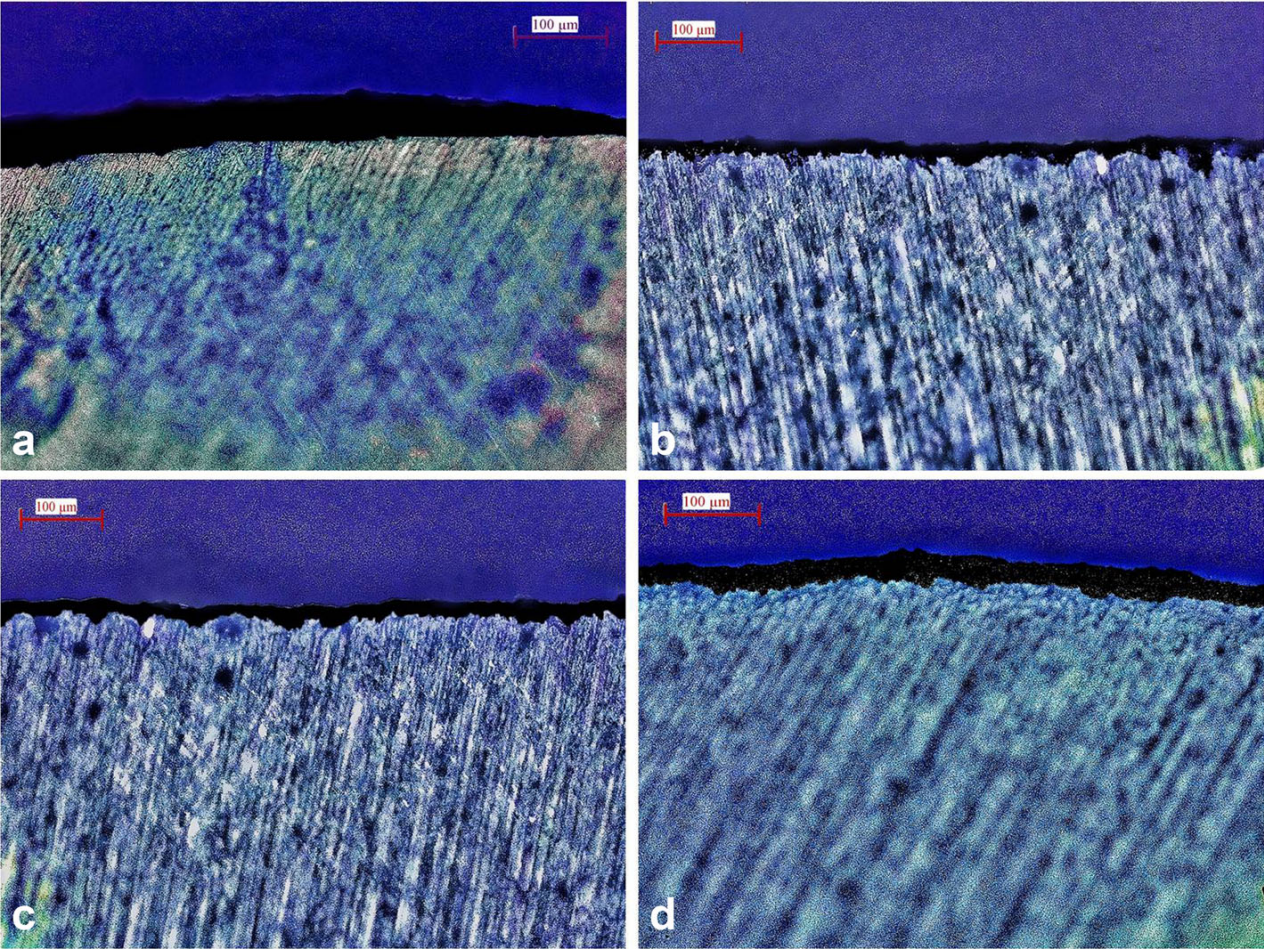
PLM images of demineralized areas for all groups (×20): **a**) No treatment (control); **b**) MI Varnish; **c**) Clinpro White; **d**) Duraphat

Means and standard deviations of lesion depth values are given in Table 3, and the demineralized areas for all groups were represented in Fig. 1. Mean lesion depth was lowest in the MI varnish group and greatest in the no treatment (control) group and, the differences in mean lesion depth measurements between groups were statistically significant (*P* < 0.05).

**Table 3:** Means and standard deviations of lesion depth values

Group	Mean ± Standard Deviation (μm)
No treatment (control)	54.56 ± 4.16^d^
MI Varnish	23.60 ± 3.36^a^
Clinpro White	29.85 ± 3.27^b^
Duraphat	40.37 ± 3.41^c^

Different superscript letters indicate statistically significant differences (one-way ANOVA, Tukey’s test, *P* < 0.05, *n* = 10 molars in each group)

**Fig. 2:**
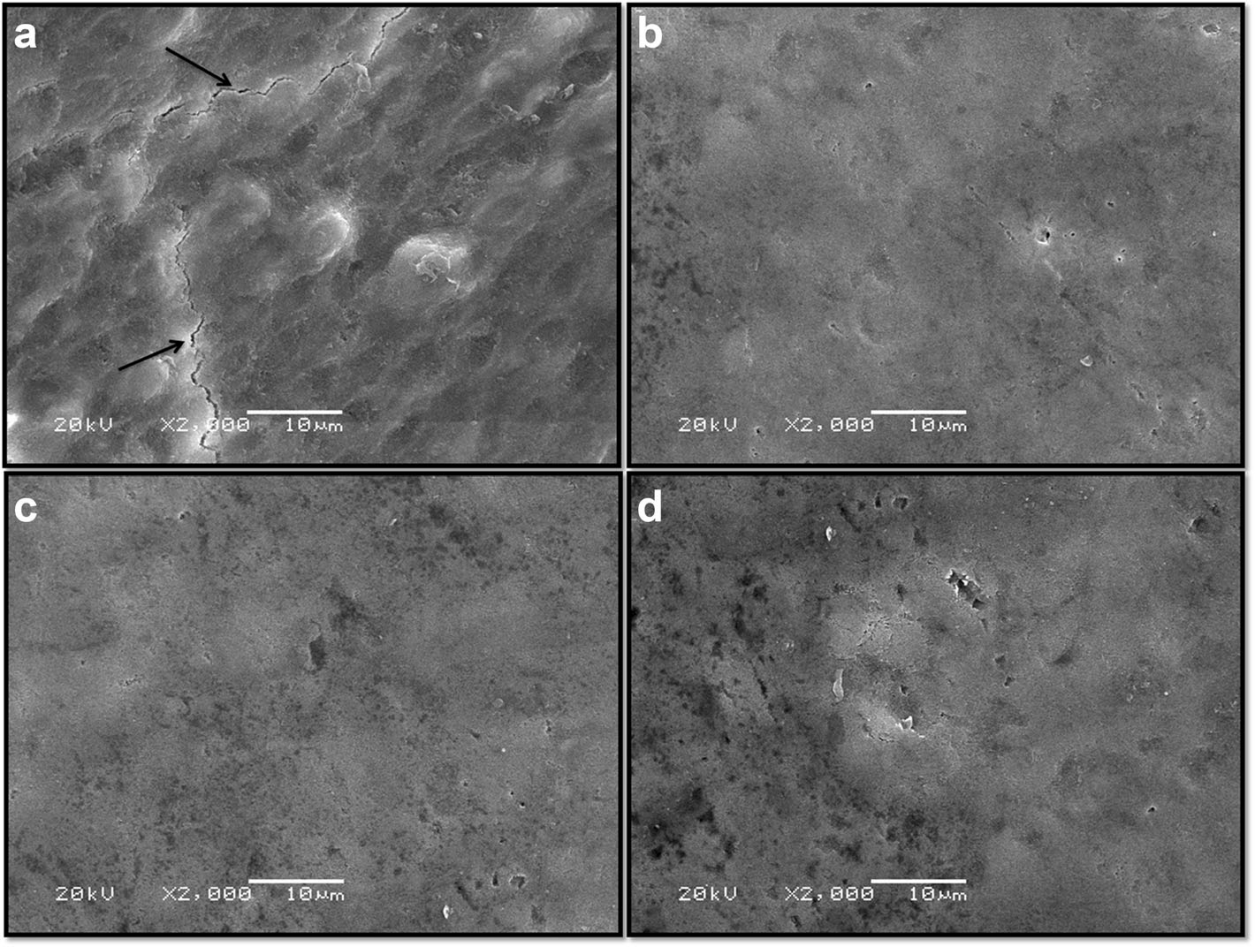
SEM images of enamel surfaces for all groups (×2000): **a**) No treatment (control) (*arrows represent crack); **b**) MI Varnish; **c**) Clinpro White; **d**) Duraphat

SEM images of enamel surfaces are shown in Fig. 2. The enamel prisms were clearly visible on the SEM images in no treatment (control) group. Furthermore, cracks were observed in control group. However, the enamel surface presents a smooth surface in the Duraphat, Clinpro White and MI varnish groups.

## Discussion

New materials are continually being introduced into dental practice. Not only do these materials require examination to confirm the properties they claim to possess, but also to propose modifications or new associations that can contribute to their improved performance. For years the application of fluoride agents in various forms has been the most effective and frequently employed method used in the prevention of dental caries [3–5]. Recently, a new fluoride varnish containing CPP-ACP became commercially available on the dental market [8, 23]; however, to the best of our knowledge, there are no published studies reporting on the effect of this product on the acid resistance of teeth. For this reason, the aim of this study was to investigate the efficacy of fluoride varnish containing CPP-ACP on the acid resistance of primary-tooth enamel.

Our results showed that fluoride varnish with CPP-ACP provided the most resistance against acid challenge of primary teeth enamel when compared the different fluoride varnishes. Also, the lowest changes in surface microhardness values were observed in MI varnish. Schemehorn et al. [24] have also been suggested that the addition of ACP rather than tricalcium phosphate may provide an even greater increase in the ability of fluoride varnish to prevent caries. The anticariogenic mechanism of CPP-ACP may involve the incorporation of nanocomplexes into plaque and onto the tooth surface [9, 10, 13, 25]. These localized CPP-ACP nanocomplexes have been purported to buffer the activity of free calcium and phosphate ions, thereby maintaining a state of supersaturation with respect to tooth enamel, preventing enamel demineralization and promoting remineralization [9–11, 25]. Pithon et al. [23] reported that MI varnish (5 % sodium fluoride varnish with added CPP-ACP) was more effective than Duraphat (5 % sodium fluoride varnish without CPP-ACP) in reducing the depth of caries lesions around orthodontic brackets. Moreover, a recent study by Schemehorn et al. [24] found varnish containing ACP to promote significantly higher levels of fluoride deposition into enamel when compared to a varnish containing tricalcium phosphate. Cochrane et al. [8], in a study evaluating release of fluoride, calcium and inorganic phosphate ions from fluoride varnishes with different material composition (MI, Clinpro White, Enamel Pro, Bifluorid 5, Duraphat), found calcium and fluoride ion release to be highest with MI; furthermore, no inorganic phosphate ion release was observed with either Bifluorid 5 or Duraphat. Previous SEM studies [12, 13] have reported a homogeneous remineralized layer on enamel surfaces treated with CPP-ACP paste.

The present study found lesion depth and changes in surface microhardness values to be significantly lower for Clinpro White when compared to Duraphat. These findings indicate that Clinpro White offers greater protection against cariogenic challenge than Duraphat. The clinical efficiency of fluoride varnishes varies according to the chemical components of the material and its method of application [5]. Fluoride varnishes are available commercially in many different forms and concentrations. One of the most widely used is 5 % Sodium fluoride (Duraphat, Colgate-Palmolive, NSW, Australia). A fluoride varnish with added tricalcium phosphate is also currently available (Clinpro White, 3M Espe, MN, USA). Previous study has reported that the addition of tricalcium phosphate to fluoride toothpaste increased fluoride retention in both enamel and dentine and facilitated remineralization [26]. In line with our findings, a study by Alamoudi et al. [27] comparing the effects of 5 % sodium fluoride varnish with and without tricalcium phosphate on the microhardness of the primary-tooth enamel reported that the addition of tricalcium phosphate significantly improved the protective ability of the varnish.

In our study, all the fluoride varnish groups had lower mean lesion depth values and smaller changes in surface microhardness when compared to an untreated control group. The presence of fluoride in the oral environment has been reported to prevent the dissolution of hydroxyapatite on enamel surfaces and increase their resistance to acid through the formation of fluorapatite [25, 28, 29]. The preventive effect demonstrated by the topical application of fluoride has also been attributed to its demonstrated ability to form calcium fluoride [25, 28]. While calcium fluoride leaches slowly and easily when challenged by acid, it does prevent the dissolution of minerals from enamel by providing a physical barrier on the enamel surface [30] and can serve as a fluoride reservoir for additional reactions, promoting enamel remineralization by maintaining high concentrations of fluoride in the oral environment [5, 25]. Our results are in accordance with of study by Santos Lde et al. [4] that investigated the effects of different fluoride agents (gel, varnish, toothpaste) on the acid-resistance of primary-tooth enamel by measuring lesion depth, found significantly lower values for lesion depth in all the fluoride groups when compared to a control group that was not treated with fluoride.

In vitro de-remineralization studies are a well-recognized method for investigating the effects of caries-prevention strategies. In this study, artificial caries were created in primary-tooth enamel using the pH-cycling model described by ten Cate and Duijsters [21]. Because primary-tooth enamel is thinner and has a lower mineral content and higher organic content than permanent-tooth enamel [31], carious lesions progress relatively faster in primary-tooth enamel [22]. Thaveesangpanich et al. [22] and ten Cate and Duijjsters [21] found that a 10-day in vitro pH-cycling model led to the progression of carious lesions in primary teeth from enamel to dentin, and suggested 7 days to be the limit for pH-cycling of primary teeth enamel [22]. In light of this information, the specimens used in this study were subjected to pH cycling for 7 days.

Many techniques have been used to evaluate the effectiveness of various processes and agents on the acid resistance of teeth. These include the evaluation of structural changes [11–13] as well as measurements of microhardness [26, 27, 32, 33], lesion depth [4, 33], mineral loss [32] and calcium, phosphate and fluoride level [8, 26]. Microhardness measurement is a reliable and effective method for assessing the protective effects of fluoride in vitro [26]. Measurement of lesion depth is also a sensitive, precise, reliable and accurate method for evaluating increases in acid-resistance of teeth [4, 33]. In this study, both surface microhardness and lesion depth were measured, and SEM analysis was used to evaluate structural changes and confirm the findings for surface microhardness and lesion depth.

## Conclusion

Within the limitations of the present study, different fluoride varnishes were all found to have positive effects in the prevention of enamel demineralization in primary teeth, with the fluoride varnish containing CPP-ACP the most effective in increasing the acid resistance of primary enamel. In order to confirm the data obtained from this in-vitro study and investigate the effects of fluoride varnish containing CPP-ACP under clinical situations in pediatric dentistry, clinical trials are required.

## Abbreviations

%VHN: Percentage of hardness loss
°C: Degree celsius or degree centigrade
CPP-ACP: Casein phosphopeptide-amorphous calcium phosphate
g: Gram
h: Hour
kV: Kilovolt
min: Minute
mm: Millimeter
mmol/L: Millimoles/Liter
pH: Power of hydrogen
PLM: Polarized light microscope
s: Second
SEM: Scanning Electron Microscopy
SPSS: Statistical Package for the Social Sciences
VHN: Vickers hardness number
VHN1: Baseline Vickers hardness number
VHN2: Post- pH cycling Vickers hardness number
μm: Micrometer

## References

[1] Selwitz RH, Ismail AI, Pitts NB (2007). Dental caries. Lancet.

[2] Featherstone JD (2008). Dental caries: a dynamic disease process. Aust Dent J.

[3] Featherstone JD, Glena R, Shariati M, Shields CP (1990). Dependence of in vitro demineralization of apatite and remineralization of dental enamel on fluoride concentration. J Dent Res.

[4] Santos Lde M, Reis JI, Medeiros MP, Ramos SM, Araújo JM (2009). In vitro evaluation of fluoride products in the development of carious lesions in deciduous teeth. Braz Oral Res.

[5] Chu CH, Mei ML, Lo EC (2010). Use of fluorides in dental caries management. Gen Dent.

[6] Vogel GL, Buzalaf MAR (2011). Oral fluoride reservoirs and the prevention of dental caries. Fluoride and the oral environment.

[7] Cochrane NJ, Cai F, Huq NL, Burrow MF, Reynolds EC (2010). New approaches to enhanced remineralization of tooth enamel. J Dent Res.

[8] Cochrane NJ, Shen P, Yuan Y, Reynolds EC (2014). Ion release from calcium and fluoride containing dental varnishes. Aust Dent J.

[9] Reynolds EC, Cain CJ, Webber FL, Black CL, Riley PF, Johnson IH (1995). Anticariogenicity of calcium phosphate complexes of tryptic casein phosphopeptides in the rat. J Dent Res.

[10] Reynolds EC, Cai F, Shen P, Walker GD (2003). Retention in plaque and remineralization of enamel lesions by various forms of calcium in a mouthrinse or sugar-free chewing gum. J Dent Res.

[11] Poggio C, Lombardini M, Dagna A, Chiesa M, Bianchi S (1999). Protective effect on enamel demineralization of a CPP-ACP paste: an AFM in vitro study. J Dent.

[12] Oshiro M, Yamaguchi K, Takamiza T, Inage H, Watanable T, Irokawa A (2007). Effect of CPP-ACP paste on tooth mineralization: an FE-SEM study. J Oral Sci.

[13] Pai D, Bhat SS, Taranath A, Sargod S, Pai VM (2008). Use of laser fluorescence and scanning electron microscope to evaluate remineralization of incipient enamel lesions remineralized by topical application of casein phospho peptide amorphous calcium phosphate (CPP-ACP) containing cream. J Clin Pediatr Dent.

[14] Cross KJ, Huq NL, Stanton DP, Sum M, Reynolds EC (2004). NMR studies of a novel calcium, phosphate and fluoride delivery vehicle-α(S1)-casein(59–79) stabilized amorphous calcium fluoride phosphate nanocomplexes. Biomaterials.

[15] Bayrak S, Tunc ES, Sonmez IS, Egilmez T, Ozmen B (2009). Effects of casein phosphopeptide-amorphous calcium phosphate (CPP-ACP) application on enamel microhardness after bleaching. Am J Dent.

[16] Liu JF, Liu Y, Stephen HC (2006). Optimal Er:YAG laser energy for preventing enamel demineralization. J Dent.

[17] Westerman GH, Hicks MJ, Flaitz CM, Powell GL (2006). In vitro caries formation in primary tooth enamel: role of argon laser irradiation and remineralizing solution treatment. J Am Dent Assoc.

[18] Correa-Afonso AM, Ciconne-Nogueira JC, Pécora JD, Palma-Dibb RG (2010). Influence of the irradiation distance and the use of cooling to increase enamel-acid resistance with Er:YAG laser. J Dent.

[19] Delbem AC, Bergamaschi M, Sassaki KT, Cunha RF (2006). Effect of fluoridated varnish and silver diamine fluoride solution on enamel demineralization: pH-cycling study. J Appl Oral Sci.

[20] Magalhães AC, Comar LP, Rios D, Delbem AC, Buzalaf MA (2008). Effect of a 4 % titanium tetrafluoride (TiF4) varnish on demineralisation and remineralisation of bovine enamel in vitro. J Dent.

[21] ten Cate JM, Duijsters PP (1982). Alternating demineralization and remineralization of artificial enamel lesions. Caries Res.

[22] Thaveesangpanich P, Itthagarun A, King NM, Wefel JS (2005). The effects of child formula toothpastes on enamel caries using two in vitro pH-cycling models. Int Dent J.

[23] Pithon MM, Dos Santos MJ, Andrade CS, Leão Filho JC, Braz AK, de Araujo RE (2015). Effectiveness of varnish with CPP-ACP in prevention of caries lesions around orthodontic brackets: an OCT evaluation. Eur J Orthod.

[24] Schemehorn BR, Wood GD, McHale W, Winston AE (2011). Comparison of fluoride uptake into tooth enamel from two fluoride varnishes containing different calcium phosphate sources. J Clin Dent.

[25] Hicks J, Garcia-Godoy F, Flaitz C (2004). Biological factors in dental caries: role of remineralization and fluoride in the dynamic process of demineralization and remineralization (part 3). J Clin Pediatr Dent.

[26] Karlinsey RL, Mackey AC, Walker TJ, Frederick KE, Blanken DD, Flaig SM (2011). In vitro remineralization of human and bovine white-spot enamel lesions by NaF dentifrices: a pilot study. J Dent Oral Hyg.

[27] Alamoudi SA, Pani SC, Alomari M (2013). The effect of the addition of tricalcium phosphate to 5 % sodium fluoride varnishes on the microhardness of enamel of primary teeth. Int J Dent.

[28] Hicks J, Garcia-Godoy F, Flaitz C (2004). Biological factors in dental caries enamel structure and the caries process in the dynamic process of demineralization and remineralization (part 2). J Clin Pediatr Dent.

[29] Featherstone JD (2000). The science and practice of caries prevention. J Am Dent Assoc.

[30] Chow LC (1990). Tooth-bound fluoride and dental caries. J Dent Res.

[31] Rao A (2008). Principles and practices of pedodontics.

[32] Delbem AC, Brighenti FL, Oliveira FA, Pessan JP, Buzalaf MA, Sassaki KT (2009). In vitro assessment of an experimental coat applied over fluoride varnishes. J Appl Oral Sci.

[33] Queiroz CS, Hara AT, Paes Leme AF, Cury JA (2008). pH-cycling models to evaluate the effect of low fluoride dentifrice on enamel de- and remineralization. Braz Dent J.

